# Dietary eubiotics of microbial muramidase and glycan improve intestinal villi, ileum microbiota composition and production trait of broiler

**DOI:** 10.1186/s40104-024-01010-x

**Published:** 2024-04-10

**Authors:** Sungbo Cho, Shanmugam Suresh Kumar, Santiago Ramirez, Rolando Valientes, In Ho Kim

**Affiliations:** 1 Department of Animal Resource and Science, Dankook University, Cheonan, Chungnam 31116 Korea; 2 Smart Animal Bio Institute Dankook University, Cheonan, Korea; 3 DSM Nutritional Products Asia Pacific, Mapletree Business City, Singapore, 117440 Singapore

**Keywords:** Broiler, Intestinal morphology, Microbial muramidase, Precision glycan, Probiotics

## Abstract

**Background:**

Optimal gut health is important to maximize growth performance and feed efficiency in broiler chickens. A total of 1,365 one-day-old male Ross 308 broiler chickens were randomly divided into 5 treatments groups with 21 replicates, 13 birds per replicate. The present research investigated effects of microbial muramidase or a precision glycan alone or in combination on growth performance, apparent total tract digestibility, total blood carotenoid content, intestinal villus length, meat quality and gut microbiota in broiler chickens. Treatments included: NC: negative control (basal diet group); PC: positive control (basal diet + 0.02% probiotics); MR: basal diet + 0.035% microbial muramidase; PG: basal diet + 0.1% precision glycan; and MRPG: basal diet + 0.025% MR + 0.1% PG, respectively.

**Results:**

MRPG group increased the body weight gain and feed intake (*P* < 0.05) compared with NC group. Moreover, it significantly increased total serum carotenoid (*P* < 0.05) and MRPG altered the microbial diversity in ileum contents. The MRPG treatment group increased the abundance of the phylum Firmicutes, and family Lachnospiraceae, Ruminococcaceae, Oscillospiraceae, Lactobacillaceae, Peptostreptococcaceae and decreased the abundance of the phylum Campilobacterota, Bacteroidota and family Bacteroidaceae. Compared with the NC group, the chickens fed MRPG showed significantly increased in duodenum villus length at end the trial.

**Conclusion:**

In this study, overall results showed that the synergetic effects of MR and PG showed enhancing growth performance, total serum carotenoid level and altering gut microbiota composition of broilers. The current research indicates that co-supplementation of MR and PG in broiler diets enhances intestinal health, consequently leading to an increased broiler production.

## Background

Poultry producers have been preferentially using cost-effective antibiotics in large quantities since 1951 to improve growth performance and prevent diseases. However, the development of microbial resistance to these antibiotics causes health issues in the food chain from animals to humans [1]. Commercial poultry faces important problems that influence their performance and gut health [2]. Currently, the European Union has been banned the use of antimicrobial growth promoters in livestock feed [3] and so have the South Korea, China and United States. Accordingly, the necessity of establishing natural and environment-friendly replacement molecules to promote general health and growth performance in poultry production has come to the forefront of research [4]. Studies for potential substitute to antibiotics have been carried out including prebiotics [5], probiotics [6], herb extracts [7], organic acids [8], yeast hydrolysate [9], and enzymes [10]. On the above-mentioned studies these substitutes are defined as natural growth promoters. Eubiotics, which are chemicals that concentrate on the maintenance and promotion of intestine eubiosis, have emerged as a substitute term in recent times [11]. The dietary supplementation of the eubiotics in animals could promote the presence of a balanced gut microbiota, which refers to healthy gut condition and functionality.

Microbial muramidase (MR) is glycosyl hydrolytic enzymes produced by plants, animals and microorganisms with high specificity to hydrolyze peptidoglycans the major structural compounds of the bacterial cell wall [12, 13]. Peptidoglycans are complex structures, formed by repeated N-acetylmuramic acid sequences connected by β-1-4 glycosidic linkages [14]. The peptidoglycans fragments are constantly being released in the gastro-intestinal tract (GIT) and accumulate in the gut lumen [15], which results in the interference of nutrient digestion and absorption [16]. MR is able to hydrolyse the β-1-4 glycosidic linkages between with N-acetylmuramic acid and N-acetyl glucosamine into simple carbohydrates and amino acids [17]. In broiler study, MR supplementation improved ileal amino acid digestibility of crude protein and increased energy utilization [18].

Precision glycans offer a potential solution to the challenges faced by the poultry industry in maintaining animal health and addressing the demand for reduced antibiotic usage. Since they are chemically synthesized, the precision glycan has a profile of glycan chains with a degree of polymerization and glycosidic bonds specifically defined, so that they can be completely processed by the microbiota, promoting the shift towards a healthy microbiota and specific microbiome pathways [11, 19]. The PG used herein is a mixture of chemically synthesized glycan chains that have been specifically defined to modulate, or signal microbial DNA to perform beneficial functions primarily connected to nitrogen metabolism and production of short chain fatty acid pathways [11, 19]. Likewise, it is common knowledge that in certain conditions, for example, enteric challenges or diets containing low quality protein, the variety of undigested protein that reaches the distal portions of the GIT of chickens may increase. Apajalahti and Vienola [19] found that certain proteins may be fermented by cecal microbiome and generate metabolites that are detrimental for health and welfare of the poultry. PG are able to in vivo shift the microbiome metabolic pathways towards better protein utilization, improved Microbiome Protein Metabolism Index, which is identified as the ratio between the abundance of beneficial by detrimental genes connected protein metabolism [20].

The combination of eubiotics may confer benefits beyond those expressed on own. We hypothesized that inclusion of MR and PG in combination could improve growth performance and gut health in broilers. To our knowledge, no data is available about the synergistic effects of MR and PG in broilers. In the current study the positive control group was supplemented with a probiotic composed by *Bacillus licheniformis* and *Bacillus subtilis* [21], commonly used in poultry production, and previously reported to have favorable impacts, such as suppressing pathogenic bacteria, promoting nutrient digestibility, and improving gut microbiota composition. The objective of this study was to evaluate the effect of MR and PG on growth performance, apparent total tract digestibility, meat quality, organ weight, blood profile, intestinal villus length and ileum microbiota and explore the synergistic effects of MR and PG in broiler chicken compared to conventional probiotics and a negative control.

## Materials and methods

### Research design, birds, and diets

A total of 1,365 one-day-old male Ross 308 broiler chickens, initial body weight (48.22 ± 0.38 g) was randomly assigned into 5 treatment groups with 21 replicates (cages) and 13 birds per replicate. Experimental groups were as followed: NC: negative control (basal diet group); PC: positive control (basal diet + 0.02% probiotics); MR: basal diet + 0.035% microbial muramidase; PG: basal diet + 0.1% precision glycan; MRPG: basal diet + 0.025% MR + 0.1% PG, respectively. The product used in this study was obtained from a commercial company (DSM Nutritional Products Ltd., Kaiseraugst, Switzerland). The details of the supplements in each group were described in Table 1. The trial period lasted for 35 d. The broilers were fed with two growth phases: phase 1 (d 1–14) and phase 2 (d 15–35). All diets were formulated to meet or exceed the nutrient requirements recommended by the National Research Council [22] and fed in mash form (Table 2). Broiler chickens were housed in 3 floor battery cages. Room temperature was maintained at 33 ± 1 °C for the first 3 d and then gradually reduced by 3 °C a week until reaching 24 °C and maintaining for the remainder of the experiment. The relative humidity was around 60%. Broiler chickens received diet and water ad libitum. Each pen had a pan feeder with a 35-cm diameter. Water was provided by evenly spaced nipple drinkers (5 nipples per pen) positioned along the side wall of the pen. Artificial light was provided 24 h/d by the use of fluorescent lights. At d 28 to 35, chromium oxide (Cr_2_O_3_, 0.2%) was added to all diets as an indigestible marker.

**Table 1:** The group design of animal experiment

Group number	Group name	Treatment information
T1	Negative control group	A basal diet supplemented with no extra additives (NC)
T2	Positive control group	A basal diet supplemented with 0.02% commercial probiotics containing *Bacillus licheniformis* and *Bacillus subtilis* (PC)
T3	Muramidase group	A basal diet supplemented with 0.035% microbial muramidase (MR)
T4	Precision biotics group	A basal diet supplemented with 0.1% precision glycan (PG)
T5	Mixture of MR and PG group	A basal diet supplemented with 0.025% MR + 0.1% PG (MRPG)

**Table 2:** Composition and nutrient profile of the basal diets (as fed basis), %

Item	Phase 1 (d 1 to 14)	Phase 2 (d 15 to 35)
Ingredients, %		
Corn	43.63	53.78
Soybean meal	35.08	28.18
Corn gluten meal	13.00	10.00
Wheat bran	3.00	3.00
Soy oil	1.76	1.51
TCP	1.81	1.81
Limestone	0.94	0.94
Salt	0.36	0.36
Methionine (99%)	0.19	0.19
Lysine	0.03	0.03
Mineral mix^1^	0.10	0.10
Vitamin mix^2^	0.10	0.10
Total	100.00	100.00
Calculated value		
Crude protein, %	23.00	20.00
Calcium, %	1.10	1.07
Phosphorus, %	0.83	0.79
Available phosphorus, %	0.54	0.52
Lysine, %	1.26	1.06
Methionine, %	0.54	0.50
Metabolized energy, kcal/kg	3,200	3,200
FAT,%	4.45	4.32
Fiber,%	3.55	3.30
Ash,%	6.76	6.30

^1^Provided per kg of complete diet: 37.5 mg Zn (as ZnSO_4_); 37.5 mg Mn (as MnO_2_); 37.5 mg Fe (as FeSO_4_·7H_2_O); 3.75 mg Cu (as CuSO_4_·5H_2_O)

^2^Provided per kg of complete diet: 15,000 IU of vitamin A, 3,750 IU of vitamin D_3_, 37.5 IU of vitamin E, 2.55 mg of vitamin K_3_, 3 mg of thiamin, 7.5 mg of riboflavin, 4.5 mg of vitamin B_6_, 24 μg of vitamin B_12_, 51 mg of niacin, 1.5 mg of folic acid, 0.2 mg of biotin and 13.5 mg of calcium pantothenate

### Growth performance and digestibility

On d 14 and 35, chickens were weighed by pen, and feed intake was recorded to calculate body weight gain (BWG), average daily feed intake, and feed conversion ratio (FCR). From d 33 to 35, clean fecal samples were collected (without feather and feed in feces) from each pen every day, and mixed together, dried in an oven (65 °C) for 72 h, and ground to pass through a 1-mm sieve. Feed and fecal samples were analyzed for dry matter (DM) and nitrogen (N) according to the methods of AOAC International [23]. The gross energy (GE) was determined using an automatic adiabatic oxygen bomb calorimeter (Parr 6300 Calorimeter, Moline, IL, USA). Chromium concentration was determined by UV absorption spectrophotometry (UV-1201, Shimadzu, Kyoto, Japan).

The equation for calculating apparent total tract digestibility (ATTD) was as follows:

$$\mathrm{ATTD}\;(\mathrm{\%}) = (1- ((\mathrm{Nf }\times \mathrm{ Cd})/ (\mathrm{Nd }\times \mathrm{ Cf}))) \times 100,$$

where Nf = nutrient concentration in feces (% DM), Nd = nutrient concentration in diet (% DM), Cf = chromium concentration in feces (% DM), and Cd = chromium concentration in diet (% DM).

### Blood profile

At d 14 and 35, blood samples were randomly drawn from the brachial veins of (*n* = 8/replicates) using a sterile syringe and kept in K_3_EDTA (Becton, Dickinson, and Co., Franklin Lakes, NJ, USA) heparinized and nonheparinized tubes to determine the blood profile. A quantity of 4 mL of blood was collected from the wing vein and centrifuged at 4,000 r/min for 15 min, and the plasma was dispensed into a 0.5-mL Eppendorf tube and stored at –80 °C. The total serum carotenoid was measured using a photometric determination (iCheck Carotene, BioAnalyt, Germany).

### Meat quality and viscera percentage

On d 35, the collected viscera broilers (*n* = 8/replicates) were weighed to determine the viscera percentage, including the breast meat, abdominal fat, gizzard, liver, spleen, and bursa of Fabricius percentages, according to the following formula:

$$\mathrm{Viscera}\;\mathrm{percentage}\left(\mathrm{expressed}\;\mathrm{as}\;\%\;\mathrm{of}\;\mathrm{body}\;\mathrm{weight}\right)=\mathrm{viscera}\;\mathrm{weight}/\mathrm{final}\;\mathrm{body}\;\mathrm{weight}\;\times100.$$

On d 35, the collected breast meat of broilers (*n* = 8/replicates) was used to determine meat quality. Breast meat color was measured using a Minolta CR-410 Chromameter (Konica Minolta Sensing Inc., Osaka, Japan) and expressed as (L*  = lightness, a*  = redness, and b*  = yellowness) values. The pH values of each breast meat sample were measured via a glass-electrode pH meter (WTW pH 340-A, WTH Measurement Systems Inc., Ft. Myers, FL, USA). To estimate the cooking loss, raw meat samples were packed into Cryovac Cook-In Bags after weighing and cooked in a water bath at 100 °C for 30 min. Samples were cooled at room temperature for 1 h and weighed again. Cooking loss was calculated as the weight difference between the initial raw and final cooked samples. Drip loss was measured using approximately 4 g of meat sample hung in a zipper bag and stored at 4 °C. After storage, moisture on the surface of the meat slice was carefully removed and weighed at d 1, 3, 5, and 7 after the sample was taken. The initial and final weight of each sample was used to calculate drip loss. To analyze water-holding capacity (WHC), 0.2 g chicken meat sample was taken and placed in a filter paper 125-mm diameter and pressed for 3 min at 26 °C. The moisture exposure of the compressed areas was determined using a digitalized area-line sensor (MT-10S, M.T. Precision Co. Ltd., Tokyo, Japan). The ratio of water in the meat area was then calculated (a smaller ratio indicates increased WHC).

### Intestinal villus length

On d 35, the collected intestine of broilers (*n* = 8/replicates) was used to determine the intestinal villus lengths. The abdominal cavity was dissected, and the intestine was separated. Segments of the mid-duodenum, mid-jejunum, and mid-ileum were taken and rinsed with cold physiological saline (0.9% saline) immediately stored in 10% buffer formalin. A section of 5-μm from each sample was cut, inserted on a glass slide, stained with hematoxylin and eosin (H&E), and the villus length was measured under light microscope [24].

### Microbiome analysis of ileum mucosa

On d 35, 8 broilers were slaughtered from each replicate. The ileum mucosa, identified as the section of small intestine between Meckel’s diverticulum and the ileo-caeco-colic junction, was removed. Approximately 5 cm of ileum was cut from the middle of the organ and the contents manually expressed into a sterile container and ileum mucosa samples were placed in frozen storage tubes and quickly placed in dry ice. DNA extraction was performed using a QIAamp Power Fecal kit (Qiagen, Germany). High-throughput sequencing of 16S rDNA gene amplicons was performed by Mecasys Co., Ltd. (Daejeon, Republic of Korea) using a NovaSeq PE250 platform (Mecasys Co., Ltd., Daejeon, Republic of Korea). The high-quality sequences were clustered into operational taxonomic units (OTUs) at a 97% similarity level, and total OTUs were obtained. The OTUs sequences were annotated with silva 132–99 database. According to the species annotation, the alpha and beta diversity were further calculated, and the differences between groups were compared to reveal the different characteristics of microbial community structure under different treatments.

### Statistical analysis

Data analyses of growth performance, ATTD, blood profile, meat quality and intestinal villus length were performed using SPSS version 22.0 for Windows (SPSS, Chicago, IL, USA). The normality of data was initially tested using the Shapiro–Wilk test. Data were then analyzed using one-way ANOVA, and means were compared using Duncan’s multiple range test. Differences were considered statistically significant at *P* ≤ 0.05. Data are expressed as the means and pooled SEM. Alpha-diversity was determined by observing the ASVs, Chao1 index, Shannon index, and Simpson index, and Pielou_ evenness indices, which account for richness and evenness. Beta-diversity was measured using principal coordinate analysis of both unweighted UniFrac and Bray–Curtis distances. Differential taxonomic markers for each group were determined using the linear discriminant analysis effect size (LEfSe).

## Result

### Growth performance, digestibility, and serum carotenoid level

The growth performance of the broilers in each treatment group is shown in Table 3. PC group did not show statistically significant difference compared to NC group. The single supplements of MR or PG also resulted in similar growth performance to PC group. Although PC, MR, and PG groups did not show significant improvement during the trial, the dietary supplement of either MR or PG had the tendency to increase BWG and FI at phase 1 (d 1–14) and overall period (d 1–35). The dietary supplementation of MRPG significantly increased the BWG and FI (*P* < 0.05) compared with NC group at the phase 1 and overall period. However, at phase 2 (d 15–35), there was no significant difference among the groups. The results of ATTD of DM, N, and GE were shown in Table 4. The digestibility test at d 35 did not indicate any significant difference among the groups.

**Table 3:** The synergistic effect of dietary microbial muramidase and precision glycan supplementation on growth performance in broiler

Items	NC	PC	MR	PG	MRPG
d 1 to 14					
BWG, g	241 ± 6.37^b^	247 ± 3.34^ab^	244 ± 4.87^ab^	251 ± 7.27^ab^	260 ± 6.32^a^
FI, g	415 ± 6.70^b^	422 ± 5.51^ab^	420 ± 7.72^ab^	424 ± 7.52^ab^	434 ± 8.19^a^
FCR	1.740 ± 0.03	1.713 ± 0.02	1.728 ± 0.03	1.710 ± 0.04	1.682 ± 0.03
d 15 to 35					
BWG, g	1,563 ± 11.66	1,581 ± 21.69	1,607 ± 18.64	1,597 ± 14.37	1,614 ± 13.31
FI, g	2,445 ± 39.21	2,466 ± 39.84	2,487 ± 37.05	2,486 ± 31.85	2,505 ± 41.27
FCR	1.565 ± 0.02	1.561 ± 0.01	1.551 ± 0.02	1.558 ± 0.01	1.555 ± 0.02
Overall (d 1 to 35)					
BWG, g	1,803 ± 15.68^b^	1,828 ± 23.77^ab^	1,851 ± 19.61^ab^	1,849 ± 13.27^ab^	1,874 ± 12.19^a^
FI, g	2,861 ± 41.76^b^	2,888 ± 40.91^ab^	2,908 ± 36.16^ab^	2,910 ± 31.73^ab^	2,940 ± 40.26^a^
FCR	1.587 ± 0.02	1.581 ± 0.01	1.574 ± 0.02	1.575 ± 0.01	1.570 ± 0.02
Mortality	6.96 ± 0.77	6.23 ± 0.68	5.86 ± 0.77	6.23 ± 0.60	5.13 ± 0.73

*NC* Basal diet, *PC* Basal diet supplemented with 0.02% probiotics, *MR* Basal diet supplemented with 0.035% microbial muramidase, *PG* Basal diet supplemented with 0.1% precision glycan, *MRPG* Basal diet supplemented with 0.025% microbial muramidase + 0.1% precision glycan, *BWG* Body weigh gain, *FI* Feed intake, *FCR* Feed conversion ratio

^a,b^Means with different superscripts in the same row differ significantly (*P* < 0.05)

**Table 4:** The synergistic effect of dietary microbial muramidase and precision glycan supplementation on nutrient digestibility in broilers on d 35

Items, %	NC	PC	MR	PG	MRPG
Dry matter	73.14 ± 0.42	73.51 ± 0.43	74.03 ± 0.39	73.77 ± 0.37	74.20 ± 0.36
Nitrogen	70.04 ± 0.43	70.25 ± 0.45	71.04 ± 0.42	70.65 ± 0.37	71.04 ± 0.37
Energy	71.61 ± 0.46	71.95 ± 0.47	72.45 ± 0.43	72.26 ± 0.38	72.68 ± 0.40

*NC* Basal diet, *PC* Basal diet supplemented with 0.02% probiotics, *MR* Basal diet supplemented with 0.035% microbial muramidase, *PG* Basal diet supplemented with 0.1% precision glycan, *MRPG* Basal diet supplemented with 0.025% microbial muramidase + 0.1% precision glycan

Additionally, Table 5 shows the levels of serum carotenoid among the groups. The significant improvement was found in MRPG both d 14 and 35. The PC and MR supplements tended to increase the serum carotenoid level. The levels of serum carotenoids were increased (*P* < 0.05) in broilers supplemented with MRPG compared with the NC at d 14 and 35. The single supplementation of PG or MR showed numerically higher serum carotenoid level than NC and no difference compared to the PC.

**Table 5:** The synergistic effect of dietary microbial muramidase and precision glycan supplementation on carotenoid in broilers

Items, mg/L	NC	PC	MR	PG	MRPG
d 14	2.09 ± 0.22^b^	2.73 ± 0.20^ab^	2.67 ± 0.30^ab^	2.23 ± 0.21^b^	3.08 ± 0.23^a^
d 35	2.35 ± 0.17^b^	2.87 ± 0.19^ab^	2.69 ± 0.16^ab^	2.54 ± 0.15^b^	3.12 ± 0.23^a^

*NC* Basal diet, *PC* Basal diet supplemented with 0.02% probiotics, *MR* Basal diet supplemented with 0.035% microbial muramidase, *PG* Basal diet supplemented with 0.1% precision glycan, *MRPG* Basal diet supplemented with 0.025% microbial muramidase + 0.1% precision glycan

^a,b^Means with different superscripts in the same row differ significantly (*P* < 0.05)

### Meat quality, relative organ weight, and intestinal villus length

As shown in Table 6, there was no difference on the meat quality parameters such as pH, WHC, color, cooking loss, and drip loss and relative organ weight (breast muscle, liver, spleen, gizzard, and bursa of Fabricius) among the treatment groups. The villus length of intestines, duodenum, jejunum and ileum at d 35 are shown in Table 7. Compared with the NC group, the chickens fed MRPG showed significantly longer villus length of duodenum (*P* < 0.05) compared to all other groups. PC group did not show longer villus length compared to NC, both MR or PG supplemented groups tended to display longer villus. The MRPG group showed the highest villus length. The villus lengths of jejunum and ileum were not different among the groups.

**Table 6:** The synergistic effect of dietary microbial muramidase and precision glycan supplementation on organ weight and meat quality in broiler

Items	NC	PC	MR	PG	MRPG
Relative organ weight, %					
Breast muscle	17.68 ± 2.15	17.86 ± 0.51	18.57 ± 1.74	17.97 ± 0.34	18.71 ± 0.65
Liver	2.54 ± 0.12	2.59 ± 0.10	2.79 ± 0.069	2.63 ± 0.14	2.94 ± 0.12
Spleen	0.14 ± 0.006	0.17 ± 0.02	0.16 ± 0.009	0.18 ± 0.01	0.17 ± 0.005
Bursa of Fabricius	0.16 ± 0.01	0.18 ± 0.006	0.2 ± 0.02	0.21 ± 0.02	0.22 ± 0.02
Gizzard	1.54 ± 0.05	1.75 ± 0.04	1.69 ± 0.13	1.73 ± 0.06	1.66 ± 0.08
Breast muscle color					
Lightness(L*)	49.32 ± 2.20	49.5 ± 1.31	48.59 ± 1.19	50.24 ± 0.61	50.15 ± 0.77
Redness(a*)	13.18 ± 0.21	13.06 ± 0.20	12.61 ± 0.39	12.38 ± 0.86	12.3 ± 0.18
Yellowness(b*)	14.73 ± 0.60	14.79 ± 0.68	15.23 ± 0.51	15.4 ± 1.17	15.77 ± 0.82
pH value	5.66 ± 0.10	5.51 ± 0.13	5.45 ± 0.02	5.59 ± 0.07	5.42 ± 0.06
Cooking loss, %	14.21 ± 0.21	14.72 ± 2.01	16.58 ± 0.54	15.56 ± 0.91	17.01 ± 2.35
WHC, %	52.58 ± 1.27	47.36 ± 1.26	52.52 ± 1.84	47.45 ± 1.67	48.91 ± 1.44
Drip loss, %					
d 1	1.39 ± 0.15	1.43 ± 0.05	1.36 ± 0.16	1.35 ± 0.12	1.26 ± 0.06
d 3	3.72 ± 0.23	3.81 ± 0.24	3.66 ± 0.15	3.44 ± 0.14	3.54 ± 0.11
d 5	5.9 ± 0.20	6.48 ± 0.25	6.22 ± 0.30	5.89 ± 0.17	6.34 ± 0.15
d 7	7.27 ± 0.93	8.22 ± 0.70	8.15 ± 0.84	7.31 ± 0.67	7.36 ± 0.46

*NC* Basal diet, *PC* Basal diet supplemented with 0.02% probiotics, *MR* Basal diet supplemented with 0.035% microbial muramidase, *PG* Basal diet supplemented with 0.1% precision glycan, *MRPG* Basal diet supplemented with 0.025% microbial muramidase + 0.1% precision glycan

**Table 7:** The synergistic effect of dietary microbial muramidase and precision biotics supplementation on villus length of the intestines in broilers on d 35

Items, mm	NC	PC	MR	PG	MRPG
Duodenum	0.808 ± 0.05^b^	0.826 ± 0.06^b^	0.977 ± 0.10^ab^	0.918 ± 0.04^ab^	1.037 ± 0.06^a^
Jejunum	0.744 ± 0.10	0.777 ± 0.07	0.825 ± 0.11	0.800 ± 0.06	0.878 ± 0.05
Ileum	0.614 ± 0.06	0.645 ± 0.03	0.661 ± 0.06	0.653 ± 0.08	0.733 ± 0.03

*NC *Basal diet, *PC *Basal diet supplemented with 0.2% probiotics, *MR *Basal diet supplemented with 0.035% microbial muramidase, *PG *Basal diet supplemented with 0.1% precision glycan, *MRPG *Basal diet supplemented with 0.025% microbial muramidase + 0.1% precision glycan

^a,b^Means with different superscripts in the same row differ significantly (*P* < 0.05)

### Microbiome analysis of ileum mucosa

**Fig. 1:**
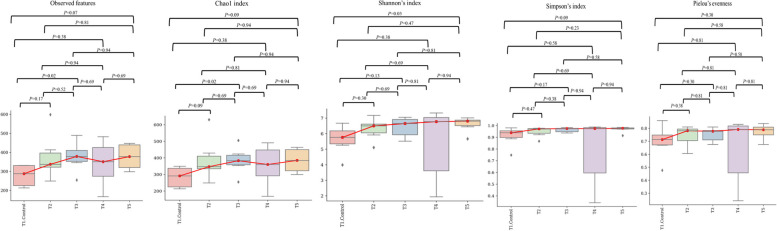
The synergistic effect of dietary microbial muramidase and precision glycans supplementation on the ileum mucosa microbiota of broilers on d 35. Species richness (Observed species and Chao) and species diversity (Shannon, Simpson and Pielous evenness). T1: negative control groups, NC (basal diet); T2: positive control groups, PC (basal diet supplemented with 0.1% probiotics); T3: muramidase groups, MR (basal diet supplemented with 0.035% microbial muramidase); T4: precision biotics groups, PG (basal diet supplemented with 0.1% precision glycan); T5: mixture of MR and PG group, MRPG groups (basal diet supplemented with 0.025% microbial muramidase + 0.1% precision glycan)

**Fig. 2:**
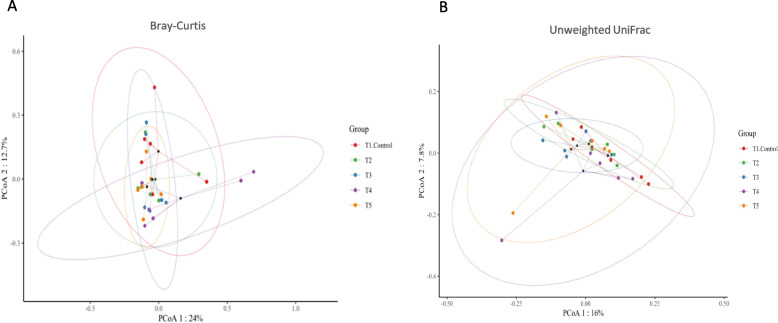
The synergistic effect of dietary microbial muramidase and precision biotics on the ileum mucosa microbiota of broilers on d 35. Principal coordinate analysis: **A** Bray Curtis and **B** unweighted UniFrac. T1: negative control groups, NC (basal diet); T2: positive control groups, PC (basal diet supplemented with 0.1% probiotics); T3: muramidase groups, MR (basal diet supplemented with 0.035% microbial muramidase); T4: precision glycan groups, PG (basal diet supplemented with 0.1% precision glycan); T5: mixture of MR and PG group, MRPG groups (basal diet supplemented with 0.025% microbial muramidase + 0.1% precision glycan)

To compare microbial diversity in each group according to the difference in dietary treatment, we performed alpha and beta diversity analysis by applying the ‘diversity’ algorithm in the QIIME2 microbiome analysis pipeline. The bacterial community classification of OTUs was performed on the basis of available sequences with 97% similarity. Microbial richness and evenness scores of each group through an alpha-diversity analysis were measured by the Observed_ ASVs, Chao 1, Shannon, Simpson, and Pielou_e α-diversity indices (Fig. 1). There was significant difference in the α-diversity of the intestinal microbiota among groups. As a result of the alpha-diversity comparison, there was no significant difference in microbial richness and evenness (in Simpson’s index and Pielou’s evenness) compared to the NC. However, the observed features and Chao1 index in ileum of broilers supplemented with MR were increased (*P* < 0.05) compared to NC and PC groups. MRPG supplemented group showed a significantly higher score in Shannon’s index, which indicates the abundance and evenness of the taxa present. Next, we performed a PERMANOVA test-based beta-diversity analysis applied with the Bray–Curtis (considering microbial abundance) (Fig. 2A) and unweighted_UniFrac (considering phylogeny) (Fig. 2B) distance matrices to confirm the dissimilarity of the estimated microbial composition among the groups. The β-diversity analysis presented overall microbial profiles of all groups. Principal coordinates analysis (PCoA) revealed that the gut microbiota in the MR, PG and MRPG groups were scattered far from the NC and PC groups. The unweighted UniFrac microbial dissimilarity was significantly found in PG and MRPG groups compare to the other groups. The PCoA results indicated that the microbiota compositions were quite dissimilar to each group and, MR, PG and MRPG supplements remarkably altered the gut microbiota composition and abundance of broilers.

**Fig. 3:**
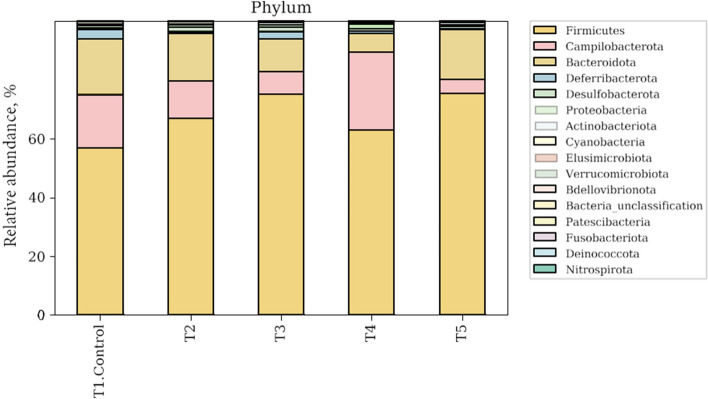
Microbial compositions of ileum microbiota in broilers at phylum levels on d 35. T1: negative control groups, NC (basal diet); T2: Positive control groups, PC (basal diet supplemented with 0.1% probiotics); T3: muramidase groups, MR (basal diet supplemented with 0.035% microbial muramidase); T4: precision glycan groups, PG (basal diet supplemented with 0.1% precision glycan); T5: mixture of MR and PG group, MRPG groups (basal diet supplemented with 0.025% microbial muramidase + 0.1% precision glycan)

**Fig. 4:**
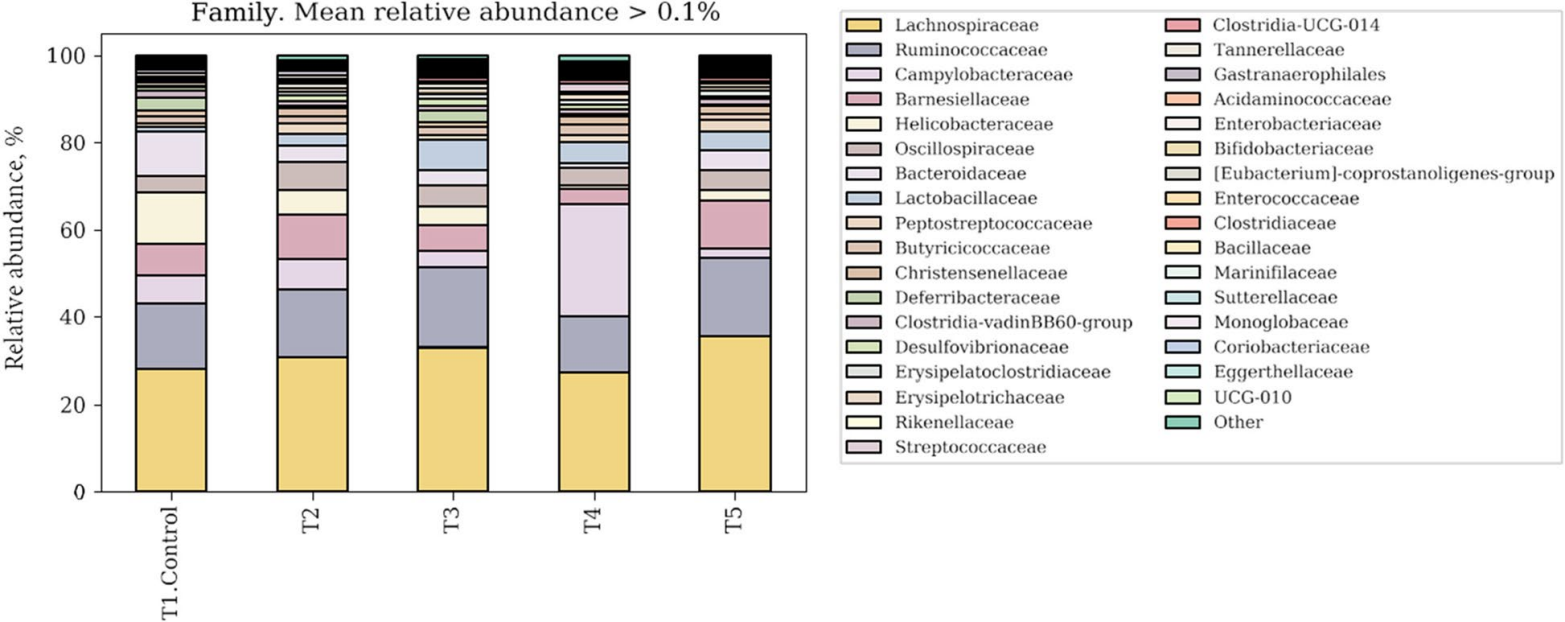
Microbial compositions of ileum microbiota in broilers at family levels on d 35. T1: negative control groups, NC (basal diet); T2: positive control groups, PC (basal diet supplemented with 0.1% probiotics); T3: muramidase groups, MR (basal diet supplemented with 0.035% microbial muramidase); T4: precision glycan groups, PG (basal diet supplemented with 0.1% precision glycan); T5: mixture of MR and PG group, MRPG groups (basal diet supplemented with 0.025% microbial muramidase + 0.1% precision glycan)

The composition of phylum (Fig. 3) and family (Fig. 4) in ileum contents of broilers are shown. The most dominant phylum of ileum microbiota were Firmicutes in all groups. At the phylum level, Firmicutes, Campilobacterota, Bacteroidota and Desulfobacterota dominated the phylum, which accounted for in excess 94%. The Firmicutes populations of NC, PC, MR, PG and MRPG treatments were 56.77%, 67.04%, 75.14%, 62.93% and 75.40%, respectively. All supplemented groups had higher Firmicutes abundance than NC group. Especially, MR and MRPG groups showed higher Firmicutes abundance than PC group. Campilobacterota populations of NC, PC, MR, PG and MRPG diets were 18.26%, 12.71%, 7.78%, 26.60% and 4.71%, respectively. Only PG supplemented group had increased Campilobacterota abundance compared to NC group. The Bacteroidota of NC, PC, MR, PG and MRPG diets were 18.95%, 16.71%, 11.04%, 6.37% and 17.15%, respectively. PC and MRPG was not much different Bacteroidota abundance compared to NC but, the Bacteroidota abundance was lower in MR and PG groups.

At the family level, the most dominant phylum of ilium microbiota was Lachnospiraceae in all groups. The microorganisms that dominated the top 6 in the NC group were Lachnospiraceae (26.03%), Ruminococcaceae (11.02%), Oscillospiraceae (3.66%), Bacteroidaceae (10.25%), Lactobacillaceae (1.07%), Peptostreptococcaceae (0.81%). NC groups showed significantly low composition of Lactobacillaceae compared to other supplemented groups. In the PC group, primarily dominated by Lachnospiraceae (30.72%), Ruminococcaceae (15.58%), Oscillospiraceae (6.22%), Bacteroidaceae (3.96%), Lactobacillaceae (2.56%), Peptostreptococcaceae (2.45%). PG group showed the highest composition of Campylobacteraceae (25.71%). The composition of Oscillospiraceae in MRPG group was higher than NC group. In the MR group, mainly dominated by Lachnospiraceae (32.93%), Ruminococcaceae (18.48%), Oscillospiraceae (5.02%), Bacteroidaceae (3.31%), Lactobacillaceae (7.07%), Peptostreptococcaceae (1.17%). In the PG group mainly dominated by Lachnospiraceae (28.93%), Ruminococcaceae (15.86%), Oscillospiraceae (3.98%), Bacteroidaceae (1.18%), Lactobacillaceae (4.63%), Peptostreptococcaceae (1.70%). In the MRPG group, mainly dominated by Lachnospiraceae (35.39%), Ruminococcaceae (18.04%), Oscillospiraceae (4.53%), Bacteroidaceae (4.38%), Lactobacillaceae (4.39%), Peptostreptococcaceae (2.70%). The composition of Peptostreptococcaceae was not significantly different among the groups except MRPG group. MRPG group indicated higher Peptostreptococcaceae abundance.

**Fig. 5:**
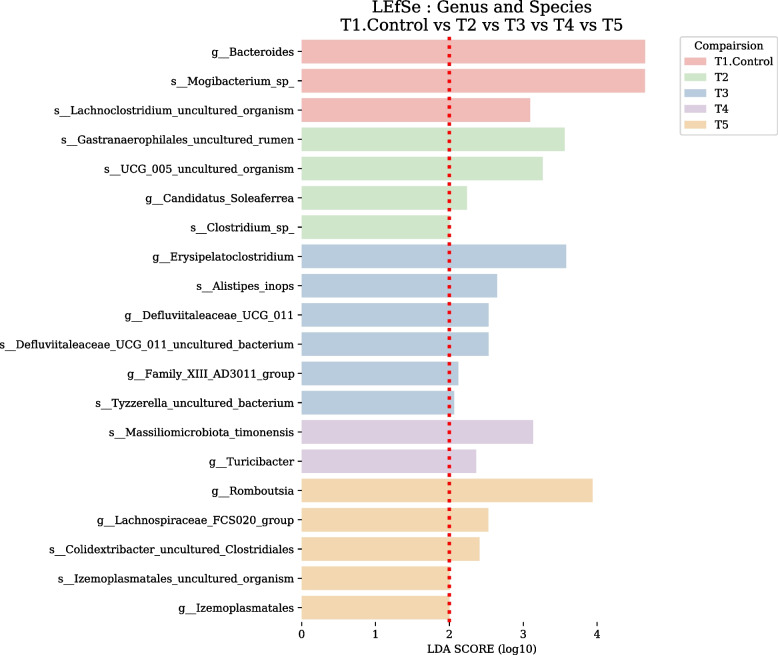
Linear discriminant analysis effect size (LEfSe) identified the most differentially abundant taxa enriched in ileum microbiota of broilers among the groups on d 35. T1: negative control groups, NC (basal diet); T2: positive control groups, PC (basal diet supplemented with 0.1% probiotics); T3: muramidase groups, MR (basal diet supplemented with 0.035% microbial muramidase); T4: precision glycan groups, PG (basal diet supplemented with 0.1% precision glycan); T5: mixture of MR and PG group, MRPG groups (basal diet supplemented with 0.025% microbial muramidase + 0.1% precision glycan)

The distinctive taxa between treatment groups were identified with LEfSe. The differential microbiota from different treatments is presented based on LEfSe analysis (Fig. 5). The findings of LDA indicated that the ileum microbiota composition was affected by feeding modification. LEfSe analysis indicated the richness of *Bacteroides, mogibacterium_*sp*,* and *Lachnoclostrdium_*uncultured_organism in PC group, *Gastranerophilales*_uncultured_organism, UCG_005_uncultured_organism, *Clostridium*_sp, and *candidatus_Soleaferrea* in PC group, *Erysipelatoclostridium, alistipes_inops*, and *Defluviitaleaceae*_UGC_011 in MR group, *massiliomicrobiota_timonensis* and *Turicibacter* in PG group, *Romboutsia, Lachnospiraceae*_FCS020_group, *Colidextribacter_uncultured_clostridiales,* and *Izemoplasmatales* in MRPG. LEfSe analysis indicated a higher number of microbiome alternations across the classes in MR and MRPG groups.

## Discussion

For poultry, probiotics could improve feed intake and digestion efficiency by increasing the activity of digestive enzymes, keep the balance of bacteria in gastrointestinal tract, promote the gut integrity and thus improve the growth performance and health of birds [25]. In addition, many studies have illustrated that probiotics be able to protect the gut barrier and modulate the gut microbiota in poultry [26–28]. The dietary supplementation of probiotics increased in growth performance of broilers [26, 29]. Similarly, Fallah et al. [21] reported that dietary inclusion of probiotic supplementation increased on growth performance of broilers. In the current study, we used probiotics as a positive control to determine the beneficial effect of eubiotics in broilers. As previously mentioned, eubiotics is known to have an essential role in supporting animal performance and animal health status by maintaining beneficial microbiota in the intestinal tract. In our result, the broilers fed PG supplement also showed improved growth performance. The biochemistry of glycans from the host and of dietary origin in the gut is exceptionally diverse. Glycans play an important role in shaping both the taxonomy and the functions of the microbiota [30, 31]. Previously, Goes et al. [32], Walsh et al. [11], and Bortoluzzi et al. [20] found that supplementation of MR and PG in broiler chickens have beneficial effect on the growth performance nutrient digestibility, apparent ileal digestibility and gut health.

In the current study, the differences of growth performance were only found at phase 1 and as a result on the overall period. The increased BWG at the early phase of broiler seems to impact on the final BWG thus, the supplementation of the MR, PG, and MRPG could be beneficial to young broiler chicks at early growth stage. In particular the combination of MR and PC showed the most significant improvements in the broiler performance during the starter phase and overall period. However, there was no significantly effect of mortality rate in MRPG groups compared to single supplement of MR or PG groups. Previously, Goes et al. [32] reported that the inclusion of MR improved the growth performance of broilers. Similarly, Lichtenberg et al. [33] and Boroojeni et al. [34] demonstrated that the supplementation of MR in diets of broilers significantly enhanced the growth performance at overall period. The hydrolyzed products of bacterial peptidoglycans (PGNs) could be reduced from the cell wall of GIT. Although our digestibility results did not show the significant improvement, we speculated the MR and MRPG supplements would result on redirecting nutrients for improving growth in broiler. Moreover, PG is known as modulator of gut microbiome metabolic pathways [11]. PG are the major structural components of the cell wall, uniquely found in bacteria, and considered as conserved products of bacterial metabolism and activity modulators in the GI tract [35]. Recently, Bortoluzzi et al. [20] and Yan et al. [36] demonstrated that PG supplementation group had a beneficial effect on performance in broiler chickens. Furthermore, Walsh et al. [11] found that BW and FCR of broilers were enhanced by PG supplementation in the diet. However, Blokker et al. [37] did not observe a significant difference in the growth performance of dietary supplementation of PG group. Moreover, in the present study, the singular supplementation of MR and PG were not significantly different but, the combination of both MRPG supplement showed a significantly higher BWG compared to NC group. FCR and ATTD at d 35 was not significantly different among the groups, but it showed certain level of tendency of improved performance. The results of villus length in the gastro-intestinal tract indicated that dietary supplement of MRPG combination increased the villus length of duodenum. The longer lengths of the duodenal villus are usually associated to increased digestive and absorptive capacity of the intestinal tract. Moreover, although we did not measure the villus length of younger broilers, it is speculated that the probiotic supplements at early phase of broiler growth could be more beneficial on the villus growth stimulation as the BWG at phase 1 in treatment group was higher. The increased growth performance at phase 1 points out to testing the enzymes activity and digestibility rate at early growth phase of broiler in a future study. Although FCR and ATTD were not statistically improved, the combination of the resulted in the higher BWG. The supplementation of MR and PG in combination exhibited synergic effects in growth performance of broilers. Similarly, Jacquier et al. [38] investigated the supplementation of a combination of xylanase and probiotics with improved growth performance in broilers. The positive performance response could have been attributed to the synergistic effect between different active ingredients in this blend by possessing antioxidant and antimicrobial activities beside the stimulant property of the digestive enzymes with subsequent positive impact on the gut microbiota and performance parameters.

In this study, the internal organ weight percentages did not statistically differ among the treatment groups. The PC, MR, PG, and MRPG groups showed an increasing trend in the percentage weight of the liver, spleen, and bursa of Fabricius compared with the NC. The MRPG indicated the highest bursa of Fabricius weight. The increased weight of the bursa is generally known to be associated to a robust immune system in poultry. Serum carotenoid-related health benefits previously were attributed mainly to their antioxidant properties, such as radical quenching [39]. The dietary supplementation with MRPG increased total serum concentration of carotenoids in broilers. Previous studies have reported that dietary supplementation with MR has positive effects on total serum concentration of carotenoids in broilers [32, 40]. Moreover, carotenoid is known to be related to digestibility but, in this study the ATTD was not different among the groups. In addition, serum carotenoids are compounds responsible for the yellow skin color in broilers and are the most prominent source of pigmentation. However, the results of the meat color did not indicate the significant differences among the groups. The absorption and accumulation of pigment in broiler tissue have been shown to be altered by many factors, such as diet composition and disease. Carotenoid absorption is negatively correlated with gut damage. The gut microbiota plays a role in the metabolism and absorption of certain nutrients. Gut damage can alter the composition and function of the microbiota, potentially affecting the bioavailability of carotenoids. Studies have shown that an increase in carotenoid concentration in plasma was associated with improved intestinal integrity [32]. In this study, we did not observe any signs of infection, perhaps explaining the lack of significant differences, however, the MRPG supplements could improve gut health and, increased the level of plasma carotenoids.

The intestinal microbiome of poultry plays a crucial role in growth performance and immune system function. Dietary eubiotic supplements could stimulate the growth of beneficial bacteria and minimize pathogenic bacteria activity in the poultry gut. Alpha diversity could serve as an indicator of the functional resilience of the intestinal microbial ecosystem, including species richness (Observed species and Chao1) and species diversity (Shannon, Simpson and Pielous evenness) [41, 42]. MR increased observed species and Chao1 index and MRPG increased Shannons index in the ileum sample of broilers compared with NC group. The results of PcoA showed that MRPG significantly changed the diversity of ileum microbiota. The ileum microflora community in all groups were evaluated based on the following levels: phylum, and family. Our results showed that Firmicutes, Campilobacterota, and Bacteroidota are the dominant phyla in the ileum of broilers, which are consistent with previous studies [36, 41]. Firmicutes phylum produces an important substance in the gut. Firmicutes contribute to the metabolism of energy materials and play a significant role in the digestion of feed [25]. MR supplement increased the abundance of Firmicutes while PG supplement reduced the abundance of Bacteroidota in the ileum of broiler chickens. Previous investigations have also found that the ileum microbiota of dietary supplemented pigs and poultry exhibits a higher abundance of Firmicutes and lower abundance of Bacteroidota when compared to the control [11]. Lactobacillaceae family and *Lactobacillus* genus belong to Firmicutes phylum. In this study, the relative frequency of the *Lactobacillus* genus was higher in MR, PG, and MRPG. Lactobacillaceae was implicated in enhanced performance, species known for their gene encoding functional abilities associated with transport and utilization of carbohydrate metabolism [36]. *Lactobacillus* in the intestinal tract of broiler is attracting attention as a major probiotic bacterial strain as the metabolites produced by them on the mucosal surface improve carcass quality to prevent gastroenteritis. Also, the *Lactobacillus* produce lactic acid, reduce the intestinal pH, and prevent the growth of harmful bacteria. Thus, changing the pH may interfere with the growth of various harmful bacterial strains. The increase of *Lactobacillus* in livestock intestines led to the growth of beneficial bacterial strains producing short-chain fatty acids (SCFAs).

The proportion of Peptostreptococcaceae family was significantly higher in the MRPG. Peptostreptococcaceae, is usually considered normal commensal bacteria, and its proportion is higher in the gut microbiota of healthy animals compared to the ones experiencing dysbiosis of the intestinal microbiota. This may indicate that Peptostreptococcaceae helps maintain gut homeostasis. Oscillospiraceae are potentially beneficial bacteria and promote the production of secondary bile acids that are known to protect against infection with *Clostridium* *difficile* [43, 44]. Nevertheless, Oscillospiraceae abundance was only increased in PC, MR, and MRPG groups compared to NC group. In addition, the dietary supplemented groups had increased abundance of Lachnospiraceae and Ruminococcaceae family. Both could be potential beneficial gut microbiota associated to positive energy metabolism through the fermentation metabolites such acetic acid, formic acid and other SCFAs [45, 46]. Campylobacteraceae was decreased in groups. In the Campylobacteraceae family, *Campylobacter jejuni* and *Campylobacter* *coli* are known to cause diarrhea by releasing toxins [47]. Moreover, PC, MR, PG and MRPG groups decreased abundance of Bacteroidaceae compared with NC group. *Bacteroides* genus, belonging to Bacteroidaceae was not statistically altered in MRPG group, but singular MR and PG supplement significantly reduced it in ileum in this study. Among dominant beneficial bacteria are several species of *Bacteroides*, which metabolize polysaccharides and oligosaccharides, providing nutrition and vitamins to the host and other intestinal microbial residents. Nevertheless, some species of *Bacteroides* may play dual beneficial and pathogenic roles based on their locations in the host, often being beneficial in the gut but opportunistic pathogens in other body locations. Therefore, *Bacteroides* could predominate in intra-abdominal infections and other infections that originate from the gut flora (i.e., perirectal abscesses, decubitus ulcers). The findings were confirmed by LEfSe analysis, which identify unique high-dimensional biomarkers for analyzed microbial communities [48]. Collectively, the microbiome results showed that MRPG supplement had an advantage in modulating the intestinal microbial community over PC, MR and PG. The combinational dietary supplement of MR and PG had an effect of significantly increasing the abundance of beneficial bacteria and decreasing in the abundance of potentially pathogenic bacteria in the intestinal tract of the broiler.

## Conclusions

Dietary supplementation of eubiotics, the combination of MR and PG supplementation improved growth performance through improving composition of gut microbiota, increasing the level of total blood carotenoid, and lengthened intestinal villus of duodenal part of the intestine. In addition, Firmicutes, Campilobacterota, Bacteroidota and Desulfobacterota phyla were dominant in both comparison groups showed a slightly increased relative proportion. Furthermore, at the family level, the most dominant family of ilium microbiota was Lachnospiraceae in both groups. The main beneficial effect of eubiotic blend to induce changes in the intestinal microbiota by selective stimulation of health-promoting bacteria. According to our findings MRPG could enhance the growth and gut health to promising alternate to antibiotic in the livestock and poultry industry in the future.

## Abbreviations

ADFI: Average daily feed intake
ATTD: Apparent total tract digestibility
BWG: Body weight gain
DM: Dry matter
FCR: Feed conversion ratio
GE: Gross energy
MR: Microbial muramidase
MRPG: Microbial muramidase and precision glycan
N: Nitrogen
NC: Negative control
PC: Positive control
PG: Precision glycan
WHC: Water-holding capacity
